# The Generalization Complexity Measure for Continuous Input Data

**DOI:** 10.1155/2014/815156

**Published:** 2014-04-10

**Authors:** Iván Gómez, Sergio A. Cannas, Omar Osenda, José M. Jerez, Leonardo Franco

**Affiliations:** ^1^Departamento de Lenguajes y Ciencias de la Computación, Universidad de Málaga, 29071 Málaga, Spain; ^2^Facultad de Matemática, Astronomía y Física, Universidad Nacional de Córdoba, 5000 Córdoba, Argentina

## Abstract

We introduce in this work an extension for the generalization complexity measure to continuous input data. The measure, originally defined in Boolean space, quantifies the complexity of data in relationship to the prediction accuracy that can be expected when using a supervised classifier like a neural network, SVM, and so forth. We first extend the original measure for its use with continuous functions to later on, using an approach based on the use of the set of Walsh functions, consider the case of having a finite number of data points (inputs/outputs pairs), that is, usually the practical case. Using a set of trigonometric functions a model that gives a relationship between the size of the hidden layer of a neural network and the complexity is constructed. Finally, we demonstrate the application of the introduced complexity measure, by using the generated model, to the problem of estimating an adequate neural network architecture for real-world data sets.

## 1. Introduction

Feed-forward neural networks trained by back-propagation have become a standard technique for classification and prediction tasks given their good generalization properties. However, the process of selecting adequate neural network architecture for a given problem is still a controversial issue. Several important contributions regarding the number of hidden neurons needed to implement a given function in a neural architecture have been made using different methods. Baum and Haussler [1] obtained some bounds on the number of neurons in an architecture related to the number of training examples that can be learnt using networks composed of linear threshold networks. Barron [2] made an important contribution about the approximation capabilities of feed-forward networks, computing an estimation of the number of hidden nodes necessary to optimize the approximation error. Camargo and Yoneyama [3] obtained a result for estimating the number of nodes needed to implement a function using Chebyshev polynomials and previous results from Scarselli and Chung Tsoi [4] about the number of nodes needed for approximating a given function by polynomials. Hunter et al. [5] focused on the importance of selecting the learning algorithm to train closer to optimal architectures. Methods based on the geometry of output classes [6–8], single value decomposition [9], information entropy [10], and the signal to noise ratio [11] have been used to obtain an approximation to the size of hidden layer in a neural architecture.

Some of the previous studies tried to determine the adequate architecture depending on the complexity of the data set available for a given problem, but as expected measuring the complexity of data is a difficult task. Firstly, it has to be clearly defined what exactly the measure tries to quantify, as complexity can be related to several aspects of the data. Even if different complexity measures related to the size of the architectures needed to implement the data or to the complexity of learning have been proposed in the past [12–14], they have not been applied to the neural network architecture selection problem, in principle because they have not been proposed with this focus.

Moreover, several approaches have been proposed within the learning theory area to analyze the relationship between generalization and complexity. Ho et al. [15, 16] studied the complexity that characterizes the difficulty of a classification problem, and they suggest using this value to guide the selection of classifier. Sánchez et al. [17] tried to characterize the behavior of the k-NN rule when working under certain situations. More specifically, their analysis focused on the use of some data complexity measures to describe class overlapping, feature space dimensionality, and class density and discover their relation with the practical accuracy of this classifier. Duch et al. [18] suggested that the identification of datasets with high complexity is important to test new methods in computational intelligence.

But most of these analyses focused on the complexity of the architectures and on the error obtained at the end of the training process rather than on the intrinsic complexity of the data. Recently, Franco and colleagues [19, 20] have proposed a complexity measure named “generalization complexity” (GC) that aims to quantify the level of generalization ability that can be expected when Boolean data are used in a classification algorithm. The measure has been also used in the process of architecture selection involved in the implementation of a neural network, as it is expected that for more complex data larger neural network architectures might be more adequate [21]. Nevertheless, the proposed measure can only be applied to Boolean input data so, in this work, the Boolean generalization complexity is first extended to the continuous input case, to then perform a series of tests to validate the proposal using a set of continuous functions with parametrized complexity. Also, by using the set of orthonormal Walsh functions, we extend the proposal for its use with patterns of data. Finally, a model is built from which it is possible to estimate the adequate feed-forward neural network architecture for real-world benchmark data sets by choosing the number of neurons to include in the hidden layer, as the size of the input and output layers is determined by the problem.

## 2. The Generalization Complexity Measure and Its Extension to Real Input Values

Our main goal in this work is to extend the GC measure defined in *f* : {0,1}^*D*^ → {0,1} for real input and real output functions *f* : [0,1]^*D*^ → [−1,1]. The choice of the intervals [0,1] for the input and [−1,1] for the output is arbitrary and it is used for simplicity with no restrictions for the general case. We will analyze the more general case of having a continuous output as this case can later be easily particularized to the Boolean output case, more related to classification problems.

The original definition of the GC measure [19, 20] comprises two terms accounting for the first and second nearest neighbor pairs of input data points ({*e*_*i*_}), where the neighborhood is defined in terms of their Hamming distance. Let *N*_ex_ be the total number of examples (or equivalently patterns) considered and *N*_neigh_ the number of first nearest neighbors that every example (*e*_*i*_, *f*(*e*_*i*_)) has; that is, examples that are the closest Hamming distance. The first term of the GC measure, *C*_1_, known to be the more influential, is defined in Boolean space as

(1) $$\begin{array}{c} C_{1}\left[f\right]=\frac{1}{N_{\text{ex}}N_{\text{neigh}}}\overset{N_{\text{ex}}}{\underset{j=1}{\sum }}\left(\overset{}{\underset{\text{Hamming}(e_{i},e_{j})=1}{\sum }}\left|f\left(e_{i}\right)-f\left(e_{j}\right)\right|\right), \end{array}$$

where the first factor is a normalization one taking into account the number of pairs considered. Essentially, (1) measures the proportion of neighboring pairs that have different output, that is, belong to different output classes.

In the previous equation, the distance between pairs of inputs is measured by the Hamming distance, but this measure is not applicable for real valued input data. Instead, we will opt for a straightforward choice and use the Euclidean distance. We consider first the 1-dimensional (1D) case corresponding to a single continuous input variable, starting the process by discretizing the input interval [0,1] in *N* subintervals of length *h* = 1/*N*. In this way a data point, *e*_*i*_, will be indicated by the subinterval in which its coordinates are included (*x*_*i*−1_, *x*_*i*_], where *x*_*i*_ = *ih* (*i* = 1,2,…, *N*), with *x*_0_ = 0 and *x*_*N*_ = 1. The total number of examples in the 1D case is equal to *N*, while, for an arbitrary dimension *D*, the discretization of every variable in the same way leads to *N* ^*D*^ examples.

Let us define *f*_*i*_ for 1D as the value of the function at the center of subinterval *i*: *f*_*i*_ ≡ *f*((*x*_*i*−1_ + *x*_*i*_)/2), and also we assume that *d*(*e*_*i*_, *e*_*j*_) ≡ |*x*_*i*_ − *x*_*j*_| and *d*_min⁡_ = min⁡{*d*(*e*_*i*_, *e*_*j*_)} = *h*. For fixed *h*, we will say that two input data points are first nearest neighbors if they are at distance *d*_min⁡_ (this would be the equivalent of Hamming distance 1 in Boolean space).

In this way, (1) can be generalized as

(2) $$\begin{array}{c} {𝒞}_{1}\left[f\right]=\frac{1}{N_{\text{ex}}N_{\text{neigh}}\Delta f}\overset{N_{\text{ex}}}{\underset{j=1}{\sum }}\left(\overset{}{\underset{d(e_{i},e_{j})=d_{\min}}{\sum }}\left|f\left(e_{i}\right)-f\left(e_{j}\right)\right|\right), \end{array}$$

where Δ*f* = *f*_max⁡_ − *f*_min⁡_. For *D* = 1 we can obtain the first term of the complexity measure, *𝒞*_1_[*f*], for continuous input data using a grid with *N* subintervals:

(3) $$\begin{array}{c} {𝒞}_{1}\left[f\right]=\frac{1}{2N}\overset{N}{\underset{i=1}{\sum }}\left|f_{i}-f_{i-1}\right|, \end{array}$$

where we used Δ*f* = 2, *N*_ex_ = *N*, and substituted the sum over the two neighboring pairs by a forward sum over the sites. Defining the complexity measure density *𝒞*_1_′[*f*] ≡ *𝒞*_1_[*f*]/*d*_min⁡_, we can write

(4) $$\begin{array}{c} {𝒞}_{1}^{\prime }\left[f\right]=\frac{1}{2}\overset{N}{\underset{i=1}{\sum }}\left|\frac{f_{i}-f_{i-1}}{h}\right|h, \end{array}$$

which in the limit *h* → 0 (*N* → *∞*) converges to

(5) $$\begin{array}{c} {𝒞}_{1}^{\prime }\left[f\right]⟶\frac{1}{2}\overset{1}{\underset{0}{\int }}\left|\frac{df\left(x\right)}{dx}\right|dx. \end{array}$$

In terms of notation we will use *C*_1_ for the first term of the original Boolean GC measure, *𝒞*_1_ for the discretized version for continuous functions, and *𝒞*_1_′ will denote continuous generalization complexity density (CGC).

Equation (5) will be our proposal for the first term of the GC for continuous value input data for *D* = 1. Clearly, this function will be larger for more fluctuating functions as expected. For *D* = 2, we have

(6) 𝒞1[f]=h28∑i=1N∑j=1N[|fi,j−fi−1,j|+|fi,j−fi+1,j|+|fi,j−fi,j+1|+|fi,j−fi,j−1|],

where *f*_*i*,*j*_ is the value of the function within the square with coordinates *x* = *ih*, *y* = *jh*. The previous expression can be written more compactly as

(7) $$\begin{array}{c} {𝒞}_{1}\left[f\right]=\frac{h^{2}}{4}\left(\overset{N}{\underset{i=1}{\sum }}\overset{N-1}{\underset{j=1}{\sum }}\left|f_{i,j+1}-f_{i,j}\right|+\overset{N}{\underset{j=1}{\sum }}\overset{N-1}{\underset{i=1}{\sum }}\left|f_{i+1,j}-f_{i,j}\right|\right). \end{array}$$

If *f* takes alternatively the maximum and minimum values (±1) on neighboring sites, *𝒞*_1_[*f*] = 1, taking care of counting only once the difference between neighboring sites. Defining the complexity measure density *𝒞*_1_′[*f*] ≡ *𝒞*_1_[*f*]/*d*_min⁡_ as before, and following the same steps, we get

(8) $$\begin{array}{c} {𝒞}_{1}^{\prime }\left[f\right]=\frac{1}{4}\overset{1}{\underset{0}{\int }}dx\overset{1}{\underset{0}{\int }}dy\left[\left|\frac{\partial f\left(x,y\right)}{\partial x}\right|+\left|\frac{\partial f\left(x,y\right)}{\partial y}\right|\right]. \end{array}$$

The above procedure can be straightforwardly generalized to arbitrary dimension *D* obtaining

(9) $$\begin{array}{c} {𝒞}_{1}^{\prime }\left[f\right]=\frac{1}{2D}\overset{1}{\underset{0}{\int }}dx_{1}\overset{1}{\underset{0}{\int }}dx_{2}\cdots \overset{1}{\underset{0}{\int }}dx_{D}\overset{D}{\underset{i=1}{\sum }}\left|\frac{\partial f\left(\vec{x}\right)}{\partial x_{i}}\right|. \end{array}$$

We observe that (9) is not bounded; that is, there is not a function with maximum complexity. This seems to be an intrinsic difficulty as for a real function the number of maxima and minima can grow indefinitely. In any case, (8) can be useful because it can measure complexities relative to a given function.

Along similar lines, we can build the continuous version of the second term of the complexity measure, *C*_2_. In its original version for Boolean functions this term accounts for the output difference of pair of data points located at Hamming distance 2:

(10) $$\begin{array}{c} C_{2}\left[f\right]=\frac{1}{N_{\text{ex}}N_{\text{neigh}}\Delta f}\overset{N_{\text{ex}}}{\underset{j=1}{\sum }}\left(\overset{}{\underset{d(e_{i},e_{j})=2}{\sum }}\left|f\left(e_{i}\right)-f\left(e_{j}\right)\right|\right). \end{array}$$

For the continuous case we can write, for *D* = 1,

(11) C2[f]=12∑i=1N|fi+2−fih|h=12∑i=1N|(fi+2−fi+1h)−(fi+i−fih)+2(fi+1−fih)|h.

Defining the second-order complexity density as *𝒞*_2_′[*f*] ≡ *𝒞*_2_[*f*]/*d*_min⁡_, we obtain in the *h* → 0 limit

(12) $$\begin{array}{c} {𝒞}_{2}^{\prime }\left[f\right]⟶\overset{1}{\underset{0}{\int }}\left|\frac{df\left(x\right)}{dx}\right|dx. \end{array}$$

Hence, for *D* = 1, we have that *𝒞*_2_′[*f*] = 2*𝒞*_1_′[*f*]. For *D* = 2, we have

(13) 𝒞2[f]=h28∑i=1N∑j=1N(|fi,j−fi−1,j+1|+|fi,j−fi−1,j−1|+|fi,j−fi+1,j−1|+|fi,j−fi+1,j+1|+|fi,j−fi,j−2|+|fi,j−fi,j+2|+|fi,j−fi−2,j|+|fi,j−fi+2,j|),

that in the *N* → *∞* limit leads to

(14) 𝒞2′[f]⟶14∫01dx∫01dy(|∂f(x,y)∂x+∂f(x,y)∂y|+|∂f(x,y)∂x−∂f(x,y)∂y|+|∂f(x,y)∂x|+|∂f(x,y)∂y|).

Equation (14) will be our proposal for the continuous version of the second term of the GC measure.

### 2.1. Testing the Generalization Complexity on a Set of Continuous Functions

Having introduced an extension of the complexity measure for a set of continuously distributed data (9) and (14), we now would like to test the proposal, and for that we will use a set of trigonometric functions with parametrized complexity. The set in dimension *D* is defined by

(15) $$\begin{array}{c} f_{n}^{D}\left(\vec{x}\right)=\overset{D}{\underset{j=1}{\prod }}\sin\left(2\pi nx_{j}\right), \end{array}$$

with *n* taking integer values *n* = 1,2,…, even if real values can be also considered (e.g., *n* = 1/*λ*). Dividing the *D*-dimensional hypercube by using a grid of spacing 1/2*n* leads to a function that cancels at the borders of the hypercubes of side *h* = 1/2*n*, taking alternatively the values  ±1 on nearest neighbour cells. This function is precisely the well-known parity Boolean function, having a very high complexity among the set of Boolean functions [19]. Measured by the first term of the GC measure, the parity function achieves maximum complexity of 1, and thus, given a value of the discretization spacing of *h* = 1/*N*, it makes sense to consider only values of *n* up to a maximum value *n*_max⁡_ = 1/2*h* = *N*/2.

From the definition of the first term (*𝒞*_1_′) of the continuous GC measure (CGC) (9), the complexity of the set of trigonometric functions defined by (15) can be obtained:

(16) $$\begin{array}{c} {𝒞}_{1}^{\prime }\left[f_{n}^{D}\right]=\frac{2^{D}n}{\pi^{D-1}}. \end{array}$$

We observe that the complexity of the set of functions grows linearly to *n*, which is proportional to the density of points where the function cancels, a sensitive measure of the variation of the function.

The family of functions (15) can be generalized to consider different variation indexes according to the spatial direction; namely,

(17) $$\begin{array}{c} f_{n}^{D}\left(\vec{x}\right)=\overset{D}{\underset{j=1}{\prod }}\sin\left(2\pi n_{j}x_{j}\right), \end{array}$$

where **n** = (*n*_1_, *n*_2_,…, *n*_*D*_). The complexity *𝒞*_1_′ can also be easily computed and leads to

(18) $$\begin{array}{c} {𝒞}_{1}^{\prime }\left[f_{n}^{D}\right]=\frac{2^{D}}{\pi^{D-1}}\frac{1}{D}\overset{D}{\underset{j=1}{\sum }}n_{j}. \end{array}$$

**Figure 1:**
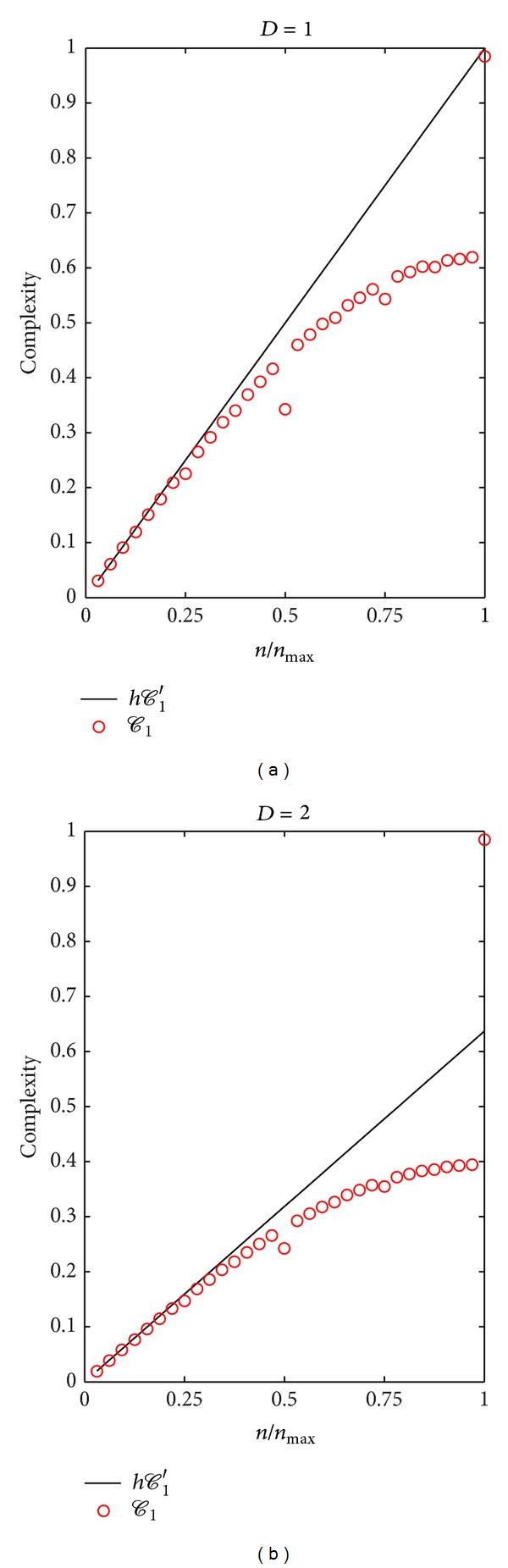
A comparison of the continuous and discrete versions of the first-order term generalization complexities for the *D* = 1 and *D* = 2 set of functions from (15) using *N* = 100. The discrete GC *C*_1_ is computed over a grid with spacing *h* and so the continuous input complexity *C*_1_′ is plotted multiplied by *h*.

We use the family of functions (15) to compare the behavior of the discrete and continuous complexity measures introduced in the previous section. To do that we computed numerically the discrete complexities *𝒞*_1_ and *𝒞*_2_ as a function of *n*/*n*_max⁡_ for *D* = 1 and 2, for a fixed value of the discretization *h*.  Figure 1 shows the complexity values obtained for the continuous and discrete first terms (*𝒞*_1_′*h* and *𝒞*_1_′, resp.) for one and two dimensions (Figures 1(a) and 1(b)), noting that for relatively low values of *n*/*n*_max⁡_, that is, when *h* ≪ 1/2*n*, the agreement is quite good, while for larger values, the discrete version underestimates the true complexity. A similar behaviour is observed for both plotted dimensions, noting that as the dimension increases the maximum complexity decreases by a factor 2^*D*^/*π* ^*D*−1^ (cf. (18)). The evaluation of the second term of the continuous complexity measure (*𝒞*_2_′) is more cumbersome but it can be obtained with the aid of numerical integration software. In particular, for *D* = 2, the calculations lead to

(19) $$\begin{array}{c} {𝒞}_{2}^{\prime }\left[f_{n}^{2}\right]=2\left(1+\frac{2}{\pi }\right)n. \end{array}$$

**Figure 2:**
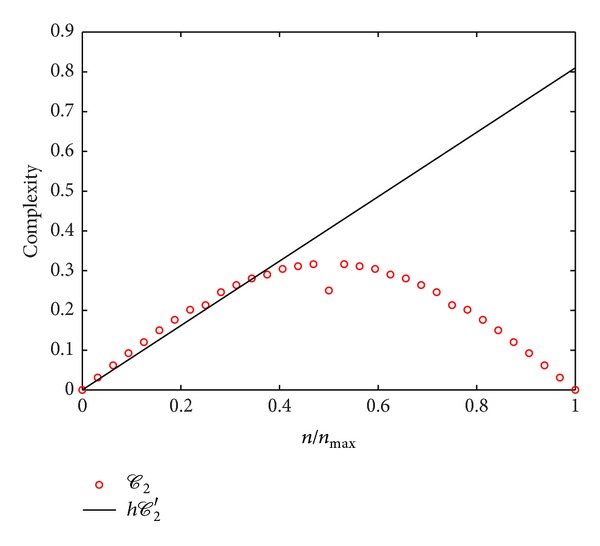
Comparison of the second terms of the complexities in their continuous and discrete versions, *h𝒞*_2_′ and *𝒞*_2_, for the two-dimensional set of trigonometric functions from (15) as a function of *n*/*n*_max⁡_.

Figure 2 shows the results for the second term of the complexity measure for the 2D set of functions. In the figure *h𝒞*_2_′[*f*_*n*_] and *𝒞*_2_[*f*_*n*_] are shown as a function of *n*/*n*_max⁡_. The continuous complexity *𝒞*_2_′ grows linearly according to what has been obtained in (19), showing a different behaviour with respect to the discrete version counterpart with a nonmonotonic curve. The quadratic-like shape of *𝒞*_2_ (in Boolean space) has been previously analyzed [19] and its behaviour independently of *𝒞*_1_ does not hold for the continuous case. The fact that the value of *𝒞*_2_′ is proportional to *𝒞*_1_′ (for the set of sinusoidal benchmark functions, cf. (15)) implies that the second term does not contain independent information from what is provided by the first term.

## 3. Use of Walsh Functions for Testing and Estimation of GC

The set of Walsh functions introduced by Walsh in 1923 [22] is a set of orthonormal binary functions with continuous input. Walsh functions have been widely applied in signal processing [23, 24] and are also well known because their relationship to the Hadamard transform [25]. The approach developed in the previous section cannot be applied to a set of patterns (the standard case for practical problems) as it requires knowing the analytic expression of the underlying function. In this section, we first compute the complexity of the set of Walsh functions showing that it leads to sensitive results for the estimation of GC. After this test, we apply the set of Walsh functions for carrying out the approximation of the GC for a set of patterns. The choice of the set of Walsh functions is motivated by the fact that the original GC defined in Boolean space can be computed almost straightforwardly for this set given its discrete output. Also, the intrinsic discretization of the input space as the order of the Walsh functions is increased favors their application to continuous input problems.

### 3.1. The GC of the Set of Walsh Functions

The proposed complexity measure (9) can be applied to the set of Walsh functions by introducing an appropriated limit procedure. Let us consider first the one-dimensional case, namely, the set of Walsh functions *W*_*n*_(*x*) defined on the real interval [0,1], where the index *n* = 0,1, 2,… is chosen so that it coincides with the number of nodes of the function. For instance, *W*_0_(*x*) = 1 for all *x*, *W*_1_(*x*) = 1 if 0 ≤ *x* < 1/2, *W*_1_(*x*) = −1 if 1/2 ≤ *x* < 1, and so forth.

**Figure 3:**
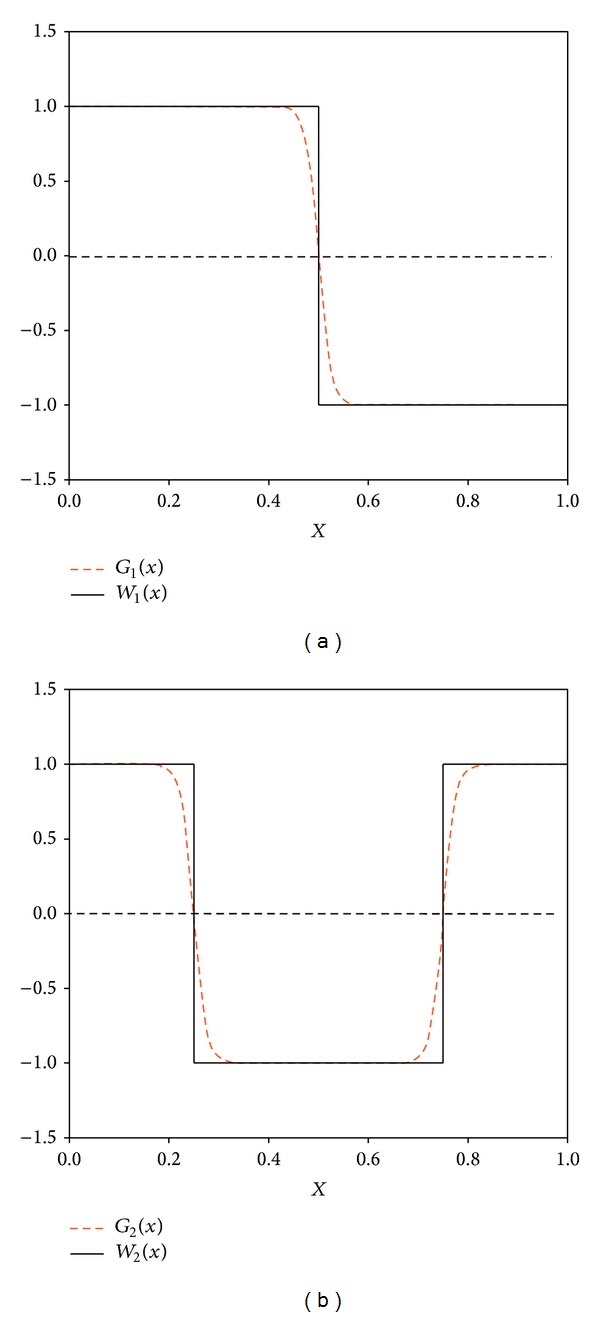
Approximation of two Walsh functions (*W*_1_(*x*) and *W*_2_(*x*)) using hyperbolic tangent functions combined with constant ones (*G*_1_(*x*) and *G*_2_(*x*)).

We will introduce a set of continuous parametric functions *G*_*n*_(*x*, *β*) to approach the Walsh functions. *G*_*n*_(*x*, *β*) can be constructed in such a way that it has the same nodes as *W*_*n*_(*x*); it is differentiable in the neighborhood of all the nodes of *W*_*n*_(*x*) and lim⁡_*β*→*∞*_ *G*_*n*_(*x*, *β*) = *W*_*n*_(*x*). The functions *G*_*n*_(*x*, *β*) can be constructed by combining sigmoidal functions centered at the nodes of *W*_*n*_(*x*) and constant functions taking values  ±1 between them, joined smoothly by any interpolation procedure, such as a spline or polynomial method.  Figure 3 shows two Walsh functions approximated by using hyperbolic tangent functions combined with constant ones.

Let us consider for simplicity a finite set of Walsh functions up to order *N* = 2^*m*^ (for some fixed integer value of *m*). Then, the location of the nodes of every one of these functions belong to the set of values *x*_i_* = *i*/*N*,  *i* = 1,2 …, *N* − 1. Let [*a*, *b*] be an arbitrary interval enclosing only one particular node *x*_i_*. Then the following properties hold:

(20) $$\begin{array}{c} \underset{\beta \to \infty }{\lim}\overset{b}{\underset{a}{\int }}\frac{\partial G_{n}\left(x,\beta \right)}{\partial x}dx=\underset{\beta \to \infty }{\lim}\left(G_{n}\left(b,\beta \right)-G_{n}\left(a,\beta \right)\right)=\pm 2,\\ \underset{\beta \to \infty }{\lim}G_{n}\left(x,\beta \right)=0\;\text{if}\;x\neq x_{i}^{*}. \end{array}$$

Hence, we can write

(21) $$\begin{array}{c} \frac{\partial G_{n}\left(x,\beta \right)}{\partial x}=2\overset{N-1}{\underset{i=1}{\sum }}g_{i}^{n}\phi \left(x-x_{i}^{*},\beta \right), \end{array}$$

where the coefficients *g*_*i*_^*n*^ can take the values 0 (if *W*_*n*_ has no node at *x*_i_*) and *g*_*i*_^*n*^ = ±1; otherwise *ϕ*(*x*, *β*) is a real function sharp peaked around *x* = 0 which satisfies lim⁡_*β*→*∞*_ *ϕ*(*x*, *β*) = *δ*(*x*), *δ*(*x*) being a Dirac delta function [26]. Then, we can define the complexity of the Walsh functions as

(22) $$\begin{array}{c} {𝒞}_{1}^{\prime }\left[W_{n}\left(x\right)\right]\equiv \underset{\beta \to \infty }{\lim}{𝒞}_{1}^{\prime }\left[G_{n}\left(x,\beta \right)\right]. \end{array}$$

From (5), (21), and (22), it follows that *𝒞*_1_′[*W*_*n*_(*x*)] = *n*. The extension to higher dimension is straightforward. Let $W_{n}(\vec{x})=\prod_{j=1}^{D}W_{n_{j}}(x_{j})$ be a D-dimensional Walsh function, where **n** = (*n*_1_,…, *n*_*D*_) is a set of one-dimensional Walsh indexes, defined as before. From (9) we obtain

(23) $$\begin{array}{c} {𝒞}_{1}^{\prime }\left[W_{n}\left(\vec{x}\right)\right]=\frac{1}{D}\overset{D}{\underset{j=1}{\sum }}n_{j}. \end{array}$$

### 3.2. GC Estimation for a Set of Data Points Using the Base of Walsh Functions

Suppose that we want to compute the coefficients, *C*_*n*_, for a given function *F* using a set of Walsh functions $W_{n}(\vec{x})$ defined in the [0,1]^*D*^

(24) $$\begin{array}{c} F\left(\vec{x}\right)≃\overset{N-1}{\underset{n=0}{\sum }}C_{n}W_{n}\left(\vec{x}\right) \end{array}$$

given a limited set of sampling data points $\left(f_{j},{\vec{x}}_{j},j=1,\ldots ,M\right)$. We will solve the estimation of the coefficients solving a minimization problem of the square error (*S*):

(25) $$\begin{array}{c} S\left(\vec{C}\right)=\overset{M}{\underset{j=1}{\sum }}{\left[f_{j}-F\left({\vec{x}}_{j}\right)\right]}^{2}=\overset{M}{\underset{j=1}{\sum }}{\left[f_{j}-\overset{N-1}{\underset{n^{\prime }=0}{\sum }}C_{n^{\prime }}W_{n^{\prime }}\left({\vec{x}}_{j}\right)\right]}^{2}, \end{array}$$

where $\vec{C}$ *≡*  (*C*_0_, *C*_1_,…, *C*_*N*−1_).

To find the minimum of the error function, *S*, we compute the first derivative and make it equal to 0:

(26) $$\begin{array}{c} \frac{\partial S}{\partial C_{n}}=-2\overset{M}{\underset{j=1}{\sum }}\left[f_{j}-\overset{N-1}{\underset{n^{\prime }=0}{\sum }}C_{n^{\prime }}W_{n^{\prime }}\left({\vec{x}}_{j}\right)\right]W_{n}\left({\vec{x}}_{j}\right)=0, \end{array}$$

from which

(27) $$\begin{array}{c} \overset{M}{\underset{j=1}{\sum }}f_{j}W_{n}\left({\vec{x}}_{j}\right)=\overset{N-1}{\underset{n^{\prime }=0}{\sum }}C_{n^{\prime }}\overset{M}{\underset{j=1}{\sum }}W_{n^{\prime }}\left({\vec{x}}_{j}\right)W_{n}\left({\vec{x}}_{j}\right). \end{array}$$

Define the vector $\vec{A}$ *≡*  (*a*_0_, *a*_1_,…, *a*_*N*−1_) as

(28) $$\begin{array}{c} a_{n}\;\;\equiv \overset{M}{\underset{j=1}{\sum }}f_{j}W_{n}\left({\vec{x}}_{j}\right) \end{array}$$

and matrix *B* = {*b*_*n*,*n*′_} with

(29) $$\begin{array}{c} b_{n,n^{\prime }}\;\;\equiv \overset{M}{\underset{j=1}{\sum }}W_{n^{\prime }}\left({\vec{x}}_{j}\right)W_{n}\left({\vec{x}}_{j}\right). \end{array}$$

Equation (27) takes the lineal form $\vec{A}$  =  $B\vec{C}$, whose solution is given by

(30) $$\begin{array}{c} \vec{C}\;\;=\;\;B^{-1}\vec{A}. \end{array}$$

A practical issue of the previous procedure is the computational cost involved; as for D-dimensional input data a matrix of size *N*_*h*_^*D*^ × *N*_*h*_^*D*^ has to be inverted (cf. (30)), where *N*_*h*_ = 1/*h* is the maximum spacing used for the construction of the 1D set of Walsh functions. Nevertheless, such computation has to be done only once for given values of *D* and *h*, being independent of the data.

Once the Walsh coefficients of a function (or data) have been obtained, the CGC can be approximated by the same limiting procedure of the previous section. For instance, in one dimension we have

(31) CGCw[F]=lim⁡β→∞𝒞1′[∑n=0N−1CnGn(x,β)]=lim⁡β→∞∫01|∑n=0N−1Cn∑i=1N−1ginϕ(x−xi∗,β)|dx=∑i=1N−1|∑n=0N−1Cngin|,

where we have used (21). For an expansion of a D-dimensional function on a finite set of *N* Walsh functions *W*_**n**_ with *n*_*j*_ = 0,1,…, *N*′ (*j* = 1,…, *D*, *N* = *N* ^′^*D*^^), we obtain similarly

(32) $$\begin{array}{c} {\text{CGC}}_{w}\left[F\right]=\frac{1}{D}\overset{N^{\prime }-1}{\underset{i=1}{\sum }}\overset{D}{\underset{j=1}{\sum }}\left|\overset{}{\underset{n}{\sum }}C_{n}g_{i}^{n_{j}}\right|, \end{array}$$

where CGC_*w*_ indicates the approximation of the CGC using the set of Walsh basis functions. We carried out an experiment where we analyzed the accuracy of the proposed approximation to obtain a similar graph to the one shown in Figure 1(a), indicating that the approximation is working correctly. The fact that the graph obtained is almost exact to the one obtained in Figure 1(a) is consistent with what can be expected, as both are discrete approximations of the continuous value of the complexity.

## 4. Application to Real-World Input Data

**Figure 4:**
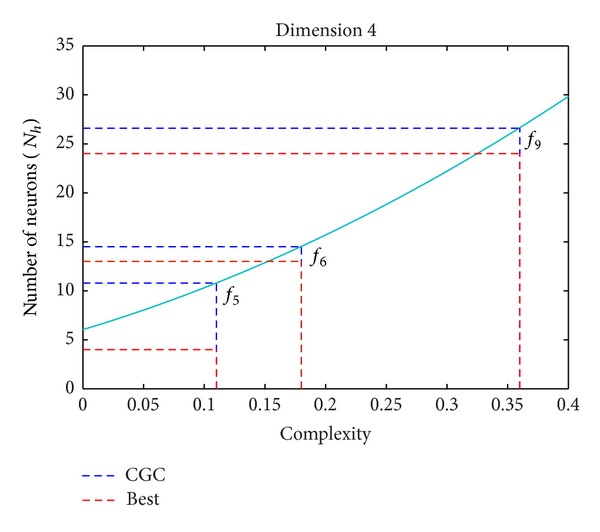
The model constructed for *N* = 4 input dimensions and its application to estimate an adequate size neural network for three test benchmark functions. The continuous line represents the model estimated from a set of trigonometric functions of variable complexity and the blue dashed line indicates the size estimated by the model (using the *Y*-axis values), while the red dashed line is the best size obtained from exhaustive numerical simulations.

In order to test practically the developed procedures, we first construct a model based on the extension of the complexity measure proposed previously, to then apply this model for the estimation of adequate neural network architecture to real-world problems. The model was estimated using the set of trigonometric functions defined by (15) for *D* = 4. For each of the analyzed data set we calculated the complexity with the above method and we found values in the range between 0 and 0.5, and the generalization ability was computed for a set of single hidden layer neural architectures with a number of neurons in the hidden layer between 2 and 50, choosing the one that leads to the lowest validation error computed in a cross-validation procedure to avoid overfitting (early stopping), where the training is performed by the standard back-propagation algorithm. From the obtained number of neurons for each of the analyzed cases, a quadratic fitting was applied to obtain the final model, shown in Figure 4 by the solid line.

Figure 4 shows the application of the developed method, described in Section 3.2, to obtain the value of CGC for a given data set. Using the constructed model (the solid line in the Figure 4), it is then possible to use the obtained CGC value to get an estimate of an adequate neural architecture to implement the function. The figure also shows the best architecture found by intensive numerical simulations (see Table 1 for the numerical values).

Table 1 shows the results obtained by applying the developed method to 10 four-dimensional benchmark data sets. The data set problems are taken from the UCI repository and for each problem 4 input variables were selected. The columns show the identifier of the function, the name of the benchmark dataset with the 4 input variables used (indicated as a superscript), the estimated Generalization complexity obtained from (22), the number of neurons in the hidden layer estimated by the model (*N*_*h*_est__), and the best number of neurons found from exhaustive simulations (*N*_*h*_Best__). The results obtained shown a quite good correlation between the estimated and best found values (*ρ* = 0.84, *P* value = 0.002), suggesting the validity of the approach, even if there are some cases, like the function indicated in the table by *f*_7_ for which the estimation is not extremely accurate. Nevertheless, some discrepancies are always expected as the problem of choosing an adequate neural architecture is a complex problem with no exact solution, as it depends on the particular set of patterns presented and the training process used, and thus it is an intrinsically noisy process.

## 5. Discussion and Conclusions

We have introduced in this work an extension for the generalization complexity (GC) measure for continuous input data. The analysis of the new measure on a parametrized complexity set of trigonometric functions shows that the new proposal is consistent with the expected results and with the spirit of the original measure, as the GC essentially measures for a set of data the output variations as the inputs are modified. Nevertheless, a difference between the continuous and discrete cases exists in relationship to the role of the second term of the GC, as in the continuous case this term is no longer independent from the first term (at least for the set of trigonometric functions), and thus it does not add extra information about the complexity of the data. We have also introduced an approach based on the use of the set of Walsh functions for computing the CGC measure for data expressed as a set of patterns, the typical case in most practical applications. By fitting a model that relates architecture size to function complexity, a model is built and then it is applied to the problem of selecting an adequate neural network architecture in ten real-world benchmark problems. The application of the method to the benchmark data shows that the estimated neural architectures are quite close to the optimal values, indicating the suitability of the developed approach to the architecture selection problem. The method is clearly more efficient than the trial-and-error alternative for choosing a proper neural network architecture, as the computationally heavy part of the procedure is related to a matrix inversion that has to be done only once for a given dimension and thus, once computed, it can be reused with different data sets. The GC measure provides an estimate of the complexity of the data, and as such can possibly be used not only for the case of choosing the adequate architecture for neural networks, but also when using other predictive models (like SVM, decision trees, etc.), for example, for choosing the magnitude of the penalization term of the model complexity (regularization).

## Figures and Tables

**Table 1:** Results of the application of the model constructed by approximating the CGC of 10 benchmark data sets from the UCI repository.

ID	Data set	CGC_*w*_	*N*_*h*_est__	*N*_*h*_Best__
*f*_1_	Balance Scale^2,3,4,5^	0.001	6.08	4
*f*_2_	Ecoli^2,4,6,7^	0.03	7.1	10
*f*_3_	Blood^1,2,3,4^	0.06	8.47	4
*f*_4_	TicTacToe^1,2,5,8^	0.1	9.84	4
*f*_5_	Liver Disorders^1,2,5,6^	0.11	10.8	4
*f*_6_	Mammografic^2,3,4,5^	0.18	14.5	13
*f*_7_	Hayes-Roth^2,3,4,5^	0.22	16.9	4
*f*_8_	Spectf^2,3,5,7^	0.26	17.8	23
*f*_9_	Vertebral Column^1,2,3,4^	0.36	26.6	24
*f*_10_	Haberman^1,2,3,4^	0.43	31.8	26

The table shows the identifier of the function, the name of the data set with superscripts indicating the 4 input used variables, the estimated CGC_*w*_, the estimated size of an adequate neural network according to the model (*N*_*h*_est__), and the best architecture found from intensive simulations (*N*_*h*_Best__).
